# Therapeutic effects and mechanisms of actions of *Descurainia sophia*

**DOI:** 10.7150/ijms.47357

**Published:** 2020-08-01

**Authors:** Po-Chun Hsieh, Chan-Yen Kuo, Yen-Hsien Lee, Yao-Kuang Wu, Mei-Chen Yang, I-Shiang Tzeng, Chou-Chin Lan

**Affiliations:** 1 Department of Chinese Medicine, Taipei Tzu Chi Hospital, Buddhist Tzu Chi Medical Foundation.; 2 School of Post-Baccalaureate Chinese Medicine, Tzu Chi University, Hualien, Taiwan.; 3 Department of Research, Taipei Tzu Chi Hospital, Buddhist Tzu Chi Medical Foundation, New Taipei City, Taiwan.; 4 Department of pulmonary medicine, Buddhist Taichung Tzu Chi General Hospital, Taichung, Taiwan.; 5 Division of Pulmonary Medicine, Taipei Tzu Chi Hospital, Buddhist Tzu Chi Medical Foundation, New Taipei City, Taiwan.; 6 School of Medicine, Tzu-Chi University, Hualien, Taiwan.

**Keywords:** *Descurainia sophia*, phytochemical effects, traditional Chinese medicine

## Abstract

*Descurainia sophia* Webb ex Prantl has been used in traditional medicine globally. It has been shown that *Descurainia sophia*, together with many other bioactive compounds, can modulate the biological functions of various genes. We have viewed the clinical benefits and mechanisms of action of *Descurainia sophia* associated with its current uses and outlined potential further applications. There are many studies documenting its numerous clinical effects in cancer, respiratory, gastrointestinal, and cardiac systems. Further, *Descurainia sophia* has been shown to exhibit anti-inflammatory, anti-oxidative, and anthelmintic activities. The clinical studies did not indicate any significant adverse effects of *Descurainia sophia*, demonstrating that it is a safe and effective herbal medicine. However, more clinical studies demonstrating the therapeutic effects of *Descurainia sophia* are still warranted.

## Introduction

Complementary and alternative medicine is popular around the world 1 and has been combined with conventional medicine to treat diverse diseases. About 38% of US and some 75% of the Republic of Korea population have been reported to use traditional medicine in their healthcare 1. Further, 76% Japanese and 93% Chinese patients used traditional medicine as complementary treatment for their diseases 1. Herbal medicine is thus an important part of complementary and alternative medicine.

*Descurainia sophia* (DS) L. Webb ex Prantl belongs to the Cruciferae (Brassiaceae) family 2. The DS plant can be found growing in temperate climate including the United States, Southern Africa, East Asia, Australia and New Zealand 2. DS has been used in herbal medicine in China, India and many other countries around the world. It has been demonstrated that DS can regulate a variety of genes closely related to numerous biological functions 3. About sixty-seven compounds have been isolated from DS and identified by gas chromatography-mass spectrometry 4. However, fifty-one compounds accounted for 98% of the total amount 4. The aerial part and seeds of DS have also found to contain several such bioactive compounds 2. Many phytochemicals have been identified from DS (aerial parts and seeds), including unique compounds of DS (descurainin, descurainin A, descurainoside, descurainoside A, and descurainoside B) that have biological activity 4-6 (**Table 1**).

Many studies addressed the effect of DS in cancer, respiratory system, gastrointestinal system, and inflammatory diseases 4-6. As evidence of pharmacological activity of DS increases, clinical uses for DS are likely to increase. Therefore, we review the use of DS herbal medicine and its mechanism of action. The known compounds of DS and their therapeutic effects are summarized in **Table 2.**

## *Descurainia sophia* activity in cancer

Despite advances in clinical medicine, the prevalence of cancer continues to rise and the disease remains the leading cause of death globally 7. Mutations in regulatory genes involved in maintaining the balance between cell proliferation and cell death lead to rapid cell growth, cell division and invasion 8. Conventional treatments of cancer such as chemotherapies or radiotherapies lack specificity. These therapies cannot distinguish normal cells from cancer cells, thus causing a great damage and death to both normal and cancer cells, resulting in significant adverse effects such as bone-marrow suppression, fatigue, gastrointestinal complications, loss of appetite, body-weight loss, etc., and often in significant negative effects on the health-related quality of life. In addition, such side effects may limit treatment programs and hence may affect the prognosis for patients. Ideally, anticancer-treatment strategy should specifically target cancer cells without affecting normal cells.

### Cytotoxic activity of *Descurainia sophia*

Many studies showed that DS possess prominent cytotoxic activity against a number of human cancer-cell lines such as lung, liver, colon, prostate, ovary, skin, and stomach 9. DS is therefore offers an option for use in cancer therapy 9. There are several constituents isolated from DS showed cytotoxic effects on cancer cell lines 3. Helveticoside has been considered as the main active cytotoxic constituent of DS 10. Quercetin also showed a potent cytotoxicity against cancer cell lines 10. Therefore, DS herb appears to show potential for the development of an anticancer supplement.

### Mechanisms of cytotoxic activity of *Descurainia sophia*

Studies are available on the DS anti-cancer mechanism of action. It has been shown that ethanolic extract of DS seeds (EEDS) has anti-cancer activity via both suppression in cancer-cell growth and cytotoxic effect 3. It is suggested that EEDS significantly inhibited cell growth by regulation of the genes involved in cell growth signaling 3. EEDS can also induce apoptotic cell death in lung cancer cells 3. The pathway of mitogen-activated protein kinase (MAPK) has been suggested to be important in the cancer invasion and growth of lung cancer 11. EEDS has been shown to up-regulate the MAPK pathway and therefore has anti-lung cancer effects 3. The other possible mechanism of anti-cancer effect of DC is that helveticoside act as anti-cancer agent by inhibiting Na^+^/K^+^ ATPase activity 12. The potential of Na^+^/K^+^ ATPase inhibitors to have anti-cancer effects has been demonstrated in many cancers such as prostate, breast, lung and leukemia 13.

**Figure 1:**
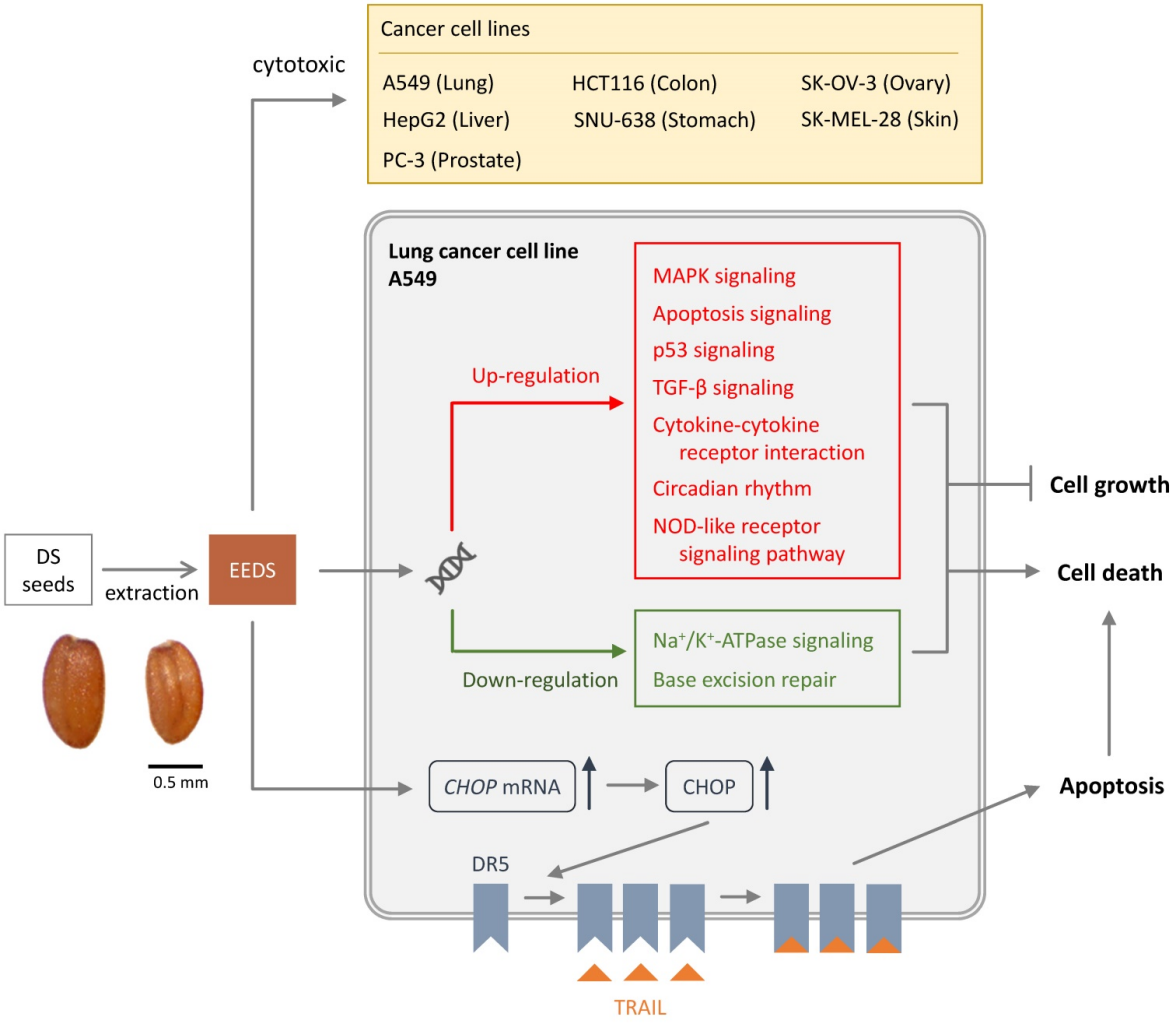
Cytotoxic effects on cancer cells of *Descurainia Sophia.*

Tumor necrosis factor (TNF)-related apoptosis-inducing ligand (TRAIL), a member of the TNF superfamily, can selectively induce cancer-cell apoptosis through the death receptors 14. As a cytotoxic cytokine, TRAIL selectively induces apoptosis in tumor cells through homotrimeric binding to the membrane bound death receptor 5 (DR5) 14. This pathway recruits Fas-related death-domain proteins and activates caspase pathway. Some cancer cells are highly malignant due to their resistance to TRAIL-induced programmed cell death. The mechanisms of TRAIL resistance include reduced expression of death receptors, overexpression of inhibitor of apoptosis, and decreased release of caspases into the cytosol 14. TRAIL resistance in cancer cells can be overcome by reversing the mechanisms of resistance, such as by upregulating death receptors 14. TRAIL is an attractive anticancer agent because it has the ability to induce apoptosis in cancer cells while sparing most of normal cells. EEDS is an appropriate herbal addition to anticancer drugs for its beneficial effect elicited by acting on the TRAIL pathway 5. It has been shown that in TRAIL-resistant cancer cells, DR5 are significantly up-regulated by EEDS 5. The mechanism by which EEDS (**Figure 1**) up-regulates DR5 is mediated by endoplasmic reticulum stress-induced transcription factors CCAAT/enhancer-binding protein homologous protein (CHOP) 5. By effectively upregulating death receptors, EEDS can sensitize TRAIL-refractory cancer cells 5.

## *Descurainia sophia* activity in respiratory diseases

DS is commonly used in traditional medicine to treat asthma and cough 4. Consequently, DS would be expected to have therapeutic effects on the respiratory system.

### Anti-Asthmatic Effects

Asthma is a multi-factorial disease determined by multiple factors such as genetic, epigenetic, and environmental 15. Although there are multiple causes of asthma, airway inflammation is an important trigger factor. In recent years, epigenetic processes regulating immune cells were recognized as playing a role in mechanisms of asthma. Allergic inflammation is the main pathogenesis of allergic asthma. The type-2 cytokines such as IL-4, IL-5, and IL-13 are important in the initiation and progression of asthma 16. The regulation of the methylation of genes related to these cytokines plays an essential role in asthma 2.

**Figure 2:**
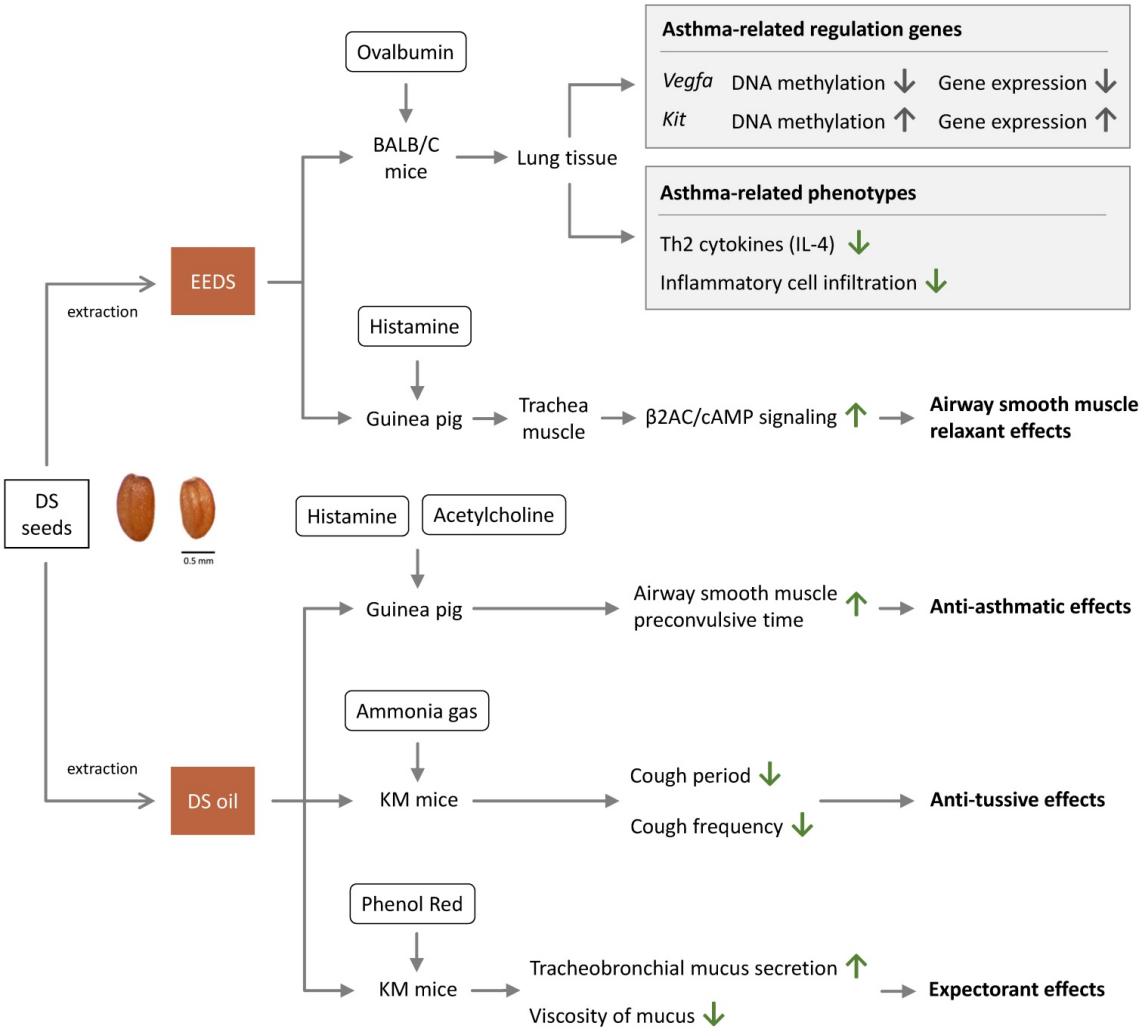
Anti-asthma effects of *Descurainia sophia.*

The mechanisms of therapeutic effects of DS are summarized in **Figure 2.** A study that examined the effects of EEDS in mouse model asthma showed that EEDS decreased infiltration of immune cells into the airways 2. This study revealed that EEDS decrease infiltration of immune cells around the airway 2. According to this study, EEDS decreased expression of Th2 cytokines and infiltration of inflammatory cell into the lungs 2. The study provided a valuable insight into the anti-asthmatic effects of DS by explaining the mechanism of traditional use of DS in treating asthma 2. The study also demonstrated that in ovalbumin (OVA)-induced asthmatic mouse model, EEDS could regulate DNA methylation and gene expression, including two functionally significant hub genes: down-regulated vascular endothelial growth factor A (*Vegfa*) and up-regulated proto-oncogene receptor tyrosine kinase (*Kit*) 2.

Another study reported that DS increased the pre-spasm time of smooth muscle induced by acetylcholine and histamine in guinea pigs 4. Descurainoside A and descurainolide B contained in EEDS were shown to have smooth-muscle relaxation effects via the β2-adrenergic receptor/cyclic adenosine monophosphate (β2AR/cAMP) signal pathway 17. The studies indicated that DS is beneficial in controlling asthma through regulating inflammatory processes and smooth-muscle relaxation.

### Anti-tussive effects

A study demonstrated that DS could prolong the latent period of cough (increasing it by 23.1%) in ammonia-liquor spray induced Kunming mice (KM mice) 4. DS could also decrease cough frequency (decreasing frequency by 15.7%) in phenol red intraperitoneal injected KM mice, thus proving the anti-tussive activities of DS *in vivo* 4.

### Expectorant effects

Gong et al. suggested that DS is able to increase tracheobronchial mucus secretion and reduce mucus viscosity, showing that DS exhibited about 40% expectorant effect as compared with control 4.

## Anti-inflammatory and anti-oxidative effects of *Descurainia sophia*

The anti-inflammatory, antipyretic, analgesic and antioxidant effects of DS were assessed in many experimental studies 6, 18, 19. Mirzaei et al. showed EEDS antioxidant effects *in vitro* 19. Nimrouzi et al. suggested that DS antioxidant effect is due to free radical scavenging 6. The antipyretic activity of DS was almost the same as that of diclofenac sodium at a dose of 400 mg/kg body weight in hyperthermic rats 18. Decoction of DS seeds was used as an antipyretic when treating smallpox and measles 20. The DS extract was also reported to have analgesic activity compared with paracetamol 18.

Quercetin and syringaresinol are active constituents of DS that impart it with anti-inflammatory effects 10. The anti-inflammatory activity of DS that were prominent especially after 2 and 3 hours of intake was ascribed to coumarins 18. Flavonoids and lignan are also DS components that inhibited nitric oxide production in lipopolysaccharides (LPS)-stimulated macrophages 10; two compounds (quercetin and syringaresinol) were isolated from DS that exerted dose-dependent inhibitory effect on NO production in LPS-stimulated RAW264.7 cells. 10 Kim et al. identified the functional involvement of DS components with responsive genes involved in the regulation of cytokine-cytokine receptor interaction and thus displaying anti-inflammatory effects 3.

## *Descurainia sophia* activity in gastrointestinal tract

Chronic constipation is a common symptom of gastrointestinal (GI) disorders. It is estimated that 12-30% of population suffers from constipation worldwide 21. DS is used as the initial treatment for constipation in traditional medicine in Iran 21. DS can act via the bowel smooth-muscle relaxation and as a stool softener 6, 9, 21. Impregnated formulations of DS seeds stimulate mucus production and absorb water from the GI lumen thus softening the stool. Allyl disulfide compound present in DS seeds are most likely acting as a smooth-muscle relaxants in the GI tract and thus promoting defecation 6, 9. Pasalar et al. found that DS could enhance bowel movement, ease defecation, and decrease abdominal distension 22. Therefore, DS is considered to offer an effective treatment for constipation and hemorrhoids 6. A clinical trial showed that a 4-weeks treatment with DS is safe (no significant adverse effects being observed) and effective in treating chronic functional constipation 21.

## *Descurainia sophia* activity in cardiac diseases

Na^+^/K^+^ ATPase is a target for the treatment of arrhythmia and congestive heart failure (CHF) 23. Cardiac glycosides have been used to treat heart failure and arrhythmia for many years 12. Cardiac glycosides exert the positive inotropic effect through inhibiting Na^+^/K^+^ ATPase activity to increase intracellular sodium, followed by inhibition of the Na^+^/Ca^2+^ exchanger to increase intracellular calcium levels for strengthening the force of the heartbeat 24. Helveticoside of DS is a cardiac glycoside and thus might have therapeutic effect in cardiac diseases 9, 12. One previous study suggested that DS could significantly increase the urinary output in the chronic heart failure of rats based on the manifestation the diuretic effect resulted from the inhibition of renal tubular to reabsorb water, sodium, and chloride 25. Zhou et al. showed that DS could decrease pleural effusion and pulmonary edema in critically ill patients 26. Another study reported that DS could improve remodeling and cardiac function in chronic heart failure rats via attenuating cardiomyocyte apoptosis by regulating the balance between Bax and Bcl-2, blocking caspase cascades with the activation of PI3k/Akt/mTOR dependent signaling 27.

## Anthelmintic activities of *Descurainia sophia*

The anthelmintic activity of DS was confirmed experimentally 6. Maraghi et al. also showed that the EEDS is effective in treating mice infected with *Hymenolepis nana*, demonstrating that administration of EEDS daily for 7 days cured mice infected with *Hymenolepis nana* 28.

## Drug-metabolizing enzyme activities of *Descurainia sophia*

Since DS is used extensively in herbal medicine, it is important to address its drug-metabolizing-enzyme (DME) activities. A study assaying enzyme activity showed that EEDS is an inhibitor with a moderate effect on CYP1A2, CYP2C9, and CYP2C19 29. Since CYP1A2, CYP2C9, and CYP2C19 are major enzymes to many vital processes, such as metabolism of endogenous compounds and elimination of environmental toxins, it is essential to consider the dosage, duration, and interactions when using DS. Many nonsteroidal anti-inflammatory drugs (NSAID) are a substrate for CYP2C9, including celecoxib, diclofenac, ibuprofen, or naproxen. As DS seeds are clinically used in the herbal formulation for anti-inflammation or treating respiratory diseases, DS seeds and NSAID will compete to bind with CYP2C9 29. The substrates for CYP1A2 include amitriptyline and erlotinib. The substrates for CYP2C9 include ibuprofen, warfarin, and tamoxifen. The substrates for CYP2C19 include diazepam, mephenytoin, methadone and bortezomib 29, 30. Clinically, the potential adverse effects of DS-drug interactions should be considered when using DS in combination with drugs that are metabolized by CYP1A2, CYP2C9, or CYP2C19 29.

## Limitations

Despite DS having a wide range of pharmacological effects and being used widely in the complementary and alternative medicine, only few clinical trials evaluated its clinical benefits. Well-designed clinical trials should be conducted to determine the DS clinical benefits and safety.

## Conclusions

**Figure 3:**
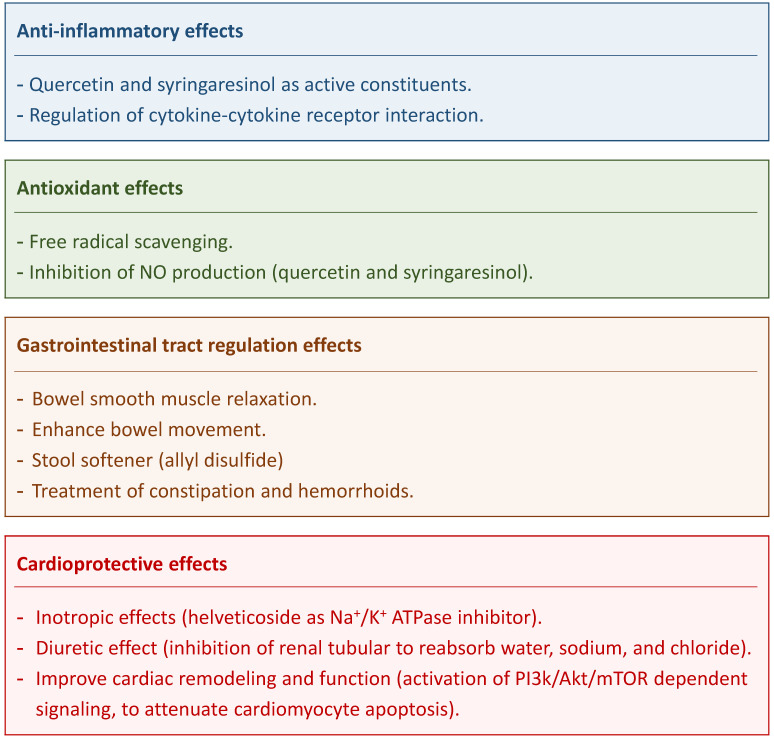
Major therapeutic effects of *Descurainia sophia.*

DS is considered to be a safe and effective herb and is commonly used in complementary and alternative medicine. In this review, we provided information suggesting that DS might be effective in cancer, respiratory diseases, gastrointestinal diseases, cardiac diseases, and other conditions (**Figure 3**). DS is rich in pharmacological constituents that could be used or developed further as therapeutic agents. While there have been many studies addressing DS' mechanisms of action, well-designed clinical trials need to be conducted to prove its clinical benefits.

## Figures and Tables

**Table 1:** Phytochemical constituents of *Descurainia sophia*

Reference	Category	Phytochemical constituents
4-6	unique compounds	descurainin, descurainin A, descurainoside, descurainoside A, descurainoside B
4-6	cardiac glycosides	erysimoside, evobioside, helveticoside, strophanthidin
4-6	coumarins	bergapten, isoscopoletin, psoralene, scopoletin, xanthotoxin, xanthotoxol
5, 6	fatty acids	arachic acid, capric acid, eicosenoic acid, erucic acid, lauric acid, linoleic acid, linolenic acid, myristic acid, oleic acid, palmitic acid, stearic acid
4-6	flavonoids	drabanemoroside, isoquercitrin, isorhamnetin, isorhamnetin-3-O-β-D-glucopyranoside, kaempferol, quercetin, quercetin 3-O-α-L-rhamnopyranosyl-(1 → 2)-α-L-arabinopyranose, quercetin-3-O-β-D-glucopyranoside
5	flavonol glycoside	artabotryside A
4, 5	glucosinolates	gluconapin, sinigrin
4-6	lactones	descurainolide A and B
5	lignan	syringaresinol
5	lipids	epoxyacylglyceride, triacylglyceride
5, 6	nor-lignan	descuraic acid
5	phenolic compounds	3,4,5-tritrimethoxy cinnamic acid, isovanillic acid, p-benzoic acid, p-hydroxybenzaldehyde, sinapic acid, syringic acid
5, 6	phytosterol	daucosterol
5	sinapoyl glycosides	1,2-di-O-sinapoyl-β-D-glucopyranose, 1,2-disinapoylgentiobiose, 1,3-di-O-sinapoyl-β-D-glucopyranose

**Table 2:** The known therapeutic effects and possible active constituents of *Descurainia sophia*

Reference	Therapeutic effects and possible active constituents of DS
3, 10	Cytotoxic effects; EEDS showed cytotoxic effects on cancer cell lines: A549 (Lung), HepG2 (Liver), PC-3 (Prostate), HCT116 (Colon), SNU-638 (Stomach), SK-OV-3 (Ovary), and SK-MEL-28 (Skin); Helveticoside is considered the primary active cytotoxic constituent.
3, 10, 18	Anti-inflammatory effects; Coumarins, flavonoids, lignan, quercetin, syringaresinol.
6, 19	Antioxidant effects; EEDS, cardiac glycoside
18	Antipyretic activity; EEDS, neoisomenthyl acetate, alloaromadendrene.
18	Analgesic activity; Benzyl, allyl, propenyl-isothiocyanate and allyl disulfide constituents.
6, 9, 21	Gastrointestinal tract regulation effects; Bowel smooth muscle relaxation; Enhance bowel movement; Stool softener (allyl disulfide); Treatment of constipation and hemorrhoids.
9, 12, 25-27	Cardioprotective effects; Inotropic effects (helveticoside as Na^+^/K^+^ ATPase inhibitor); Diuretic effect (inhibition of renal tubular to reabsorb water, sodium, and chloride); Improve cardiac remodeling and function (activation of PI3k/Akt/mTOR dependent signaling, to attenuate cardiomyocyte apoptosis).
6, 28	Anthelmintic activities; Therapeutic effects on mice infected with *Himeonolepis nana*.

## Abbreviations

β2AR: β2-adrenergic receptor
cAMP: cyclic adenosine monophosphate
CHF: congestive heart failure
CHOP: CCAAT/enhancer-binding protein homologous protein
DME: drug-metabolizing-enzyme
DS: *Descurainia sophia*
EEDS: ethanolic extract of DS seeds
GI: gastrointestinal
LPS: lipopolysaccharides
MAPK: mitogen-activated protein kinase
TNF: tumor necrosis factor
TRAIL: tumor necrosis factor-related apoptosis-inducing ligand
